# TGFBI Protein Is Increased in the Urine of Patients with High-Grade Urothelial Carcinomas, and Promotes Cell Proliferation and Migration

**DOI:** 10.3390/ijms20184483

**Published:** 2019-09-11

**Authors:** Kerstin Lang, Selcan Kahveci, Nadine Bonberg, Katharina Wichert, Thomas Behrens, Jan Hovanec, Florian Roghmann, Joachim Noldus, Yu Chun Tam, Andrea Tannapfel, Heiko U. Käfferlein, Thomas Brüning

**Affiliations:** 1 Institute for Prevention and Occupational Medicine of the German Social Accident Insurance, Institute of the Ruhr-University Bochum (IPA), Bürkle-de-la-Camp Platz 1, 44789 Bochum, Germany; lang@ipa-dguv.de (K.L.); s_kahveci@web.de (S.K.); nadinebonberg@gmx.de (N.B.); wichert@ipa-dguv.de (K.W.); behrens@ipa-dguv.de (T.B.); hovanec@ipa-dguv.de (J.H.); bruening@ipa-dguv.de (T.B.); 2 Department of Urology, Marien Hospital Herne, University Hospital of the Ruhr University Bochum, Hölkeskampring 40, 44625 Herne, Germany; Florian.Roghmann@elisabethgruppe.de (F.R.); Joachim.Noldus@elisabethgruppe.de (J.N.); 3 Institute of Pathology, Georgius Agricola Stiftung Ruhr, Ruhr University Bochum, Bürkle-de-la-Camp Platz 1, 44789 Bochum, Germany; yu.tam@rub.de (Y.C.T.); andrea.tannapfel@pathologie-bochum.de (A.T.)

**Keywords:** urothelial carcinoma, transforming growth factor beta-induced protein, TGFBI, βIGH3, proliferation, migration

## Abstract

Here, we discovered TGFBI as a new urinary biomarker for muscle invasive and high-grade urothelial carcinoma (UC). After biomarker identification using antibody arrays, results were verified in urine samples from a study population consisting of 303 patients with UC, and 128 urological and 58 population controls. The analyses of possible modifying factors (age, sex, smoking status, urinary leukocytes and erythrocytes, and history of UC) were calculated by multiple logistic regression. Additionally, we performed knockdown experiments with TGFBI siRNA in bladder cancer cells and investigated the effects on proliferation and migration by wound closure assays and BrdU cell cycle analysis. TGFBI concentrations in urine are generally increased in patients with UC when compared to urological and population controls (1321.0 versus 701.3 and 475.6 pg/mg creatinine, respectively). However, significantly increased TGFBI was predominantly found in muscle invasive (14,411.7 pg/mg creatinine), high-grade (8190.7 pg/mg) and de novo UC (1856.7 pg/mg; all *p* < 0.0001). Knockdown experiments in vitro led to a significant decline of cell proliferation and migration. In summary, our results suggest a critical role of TGFBI in UC tumorigenesis and particularly in high-risk UC patients with poor prognosis and an elevated risk of progression on the molecular level.

## 1. Introduction

Urothelial carcinoma (UC) continues to be a predominant cancer worldwide, with an estimated number of 386,000 new (de novo) cancer cases every year. Most patients are male and are diagnosed after the age of 60 [1]. At initial presentation 70%–75% of patients have a non-muscle invasive cancer (≤pT1) [2,3], of which about 50% are low-grade [4], whereas the remaining 25%–30% are muscle invasive and mostly high-grade. Disease recurrence is observed in up to 70% of patients, of whom 10%–15% experience progression to muscle invasive urothelial carcinoma [5]. Several studies found that the histological grade and stage were significant predictors of disease progression and recurrence [3,6,7]. Thus, independently from de novo or recurrent tumors, urologists are faced with the specific challenges of an early and reliable diagnosis of both high-grade and muscle invasive UCs, because of worse disease outcomes.

To date histopathological review is the only method routinely used to assess the prognosis of the patients, but is inherently subjective and known to be limited by inter-observer variability. While muscle invasive UC is histologically easily verifiable by observing the invasion of the muscularis propria bladder wall, diagnosing high-grade UC is much more challenging. According to current guidelines (WHO, 2004 [8], 2016 [9]), even tiny high-grade areas within the tissue sample are sufficient to categorize UC as high-grade. Moreover, nested variants of urothelial carcinoma are, despite their bland cytomorphology, associated with poor outcomes and controversy remains with regard to their grading, mainly whether grading should be outcome-driven [10] or solely based on their bland cytomorphological features at the time of diagnosis [4]. Similarly, controversy also remains due to the grading of pT1 tumors, superficial tumors showing infiltration of the subepithelial tissue but not being considered muscle invasive. Up to three years after initial (de novo) diagnosis, pT1 tumors show a low cancer-specific mortality (<15%) [11]. However, they also possess a high rate of recurrence and progression, and therefore, an overall poor prognosis. Consequently, the identification of biomarkers on the molecular level which are associated with progression and poor prognosis is urgently needed and a prerequisite to study whether they are better correlated with the overall clinical outcome rather than histomorphological staging or grading alone.

To better predict prognosis, much effort is spent on finding biomarkers that can be assayed easily in a reproducible manner and that specifically have convincing predictive power for UC with worse prognosis (including high-grade UC) [6]. However, at present none of the currently Food and Drug Administration (FDA)-approved biomarkers have been included into guideline recommendations or daily clinical practice, and only a few markers have been reported to discriminate high-grade UC on various molecular levels and in diverse matrices; e.g., urine or tissue. Beside urine cytology, several point of care tests measuring soluble cytokeratin fragments (UBC^®^, Rapid), nuclear matrix protein 22 (Bladder Check^®^) or complement factor H related protein (BTA TRAK^®^) are commercially available for diagnosing UC, including high-grade UC in urine [12,13,14,15,16]. In tissue, accumulation of p53 is correlated with tumor stage and grade, and as such, is observed in invasive and high-grade tumors [17]. In addition, AHNAK2 has been recently suggested as a histochemical biomarker for carcinoma in situ (pTis) [18].

In previous studies, we have identified soluble CXCL16 in patient urine as a promising biomarker for the diagnosis of high-grade UC [19]. Here, we identified soluble TGFBI and platelet-factor 4 (PF4) in urine as novel biomarker candidates for the detection of muscle invasive and high-grade UC, thereby taking personal and sample characteristics such as sex, age, presence of urinary leukocytes and erythrocytes, and smoking into account.

Transforming growth factor beta-induced (TGFBI) is an extracellular secreted matrix protein which has been proven to exist in normal and tumor cells. TGFBI has been shown to participate in various physiological processes, such as differentiation, morphogenesis, cell growth, inflammation, tumor progression and metastasis [20,21,22,23,24,25]. In many cell types TGFBI interacts with other matrix proteins, such as collagen, fibronectin and laminin, thereby mediating proliferation, migration and cell adhesion; e.g., by interacting with integrins [26,27]. Numerous cells, e.g., fibroblasts, corneal epithelial cells, smooth muscle cells and various cancer cells were demonstrated to induce TGFBI expression after transforming growth factor-β treatment, but also by interleukin-1, retinoic acid and tumor necrosis factor-α [20,28]. Accordingly, elevated TGFBI levels have been associated with a broad variety of diseases, such as corneal disorders, nephropathy [29], rheumatoid arthritis [30], cancer [24,25,26] and atherosclerosis [31]. Therefore, in addition, we investigated the function of TGFBI in vitro in the human-derived urinary bladder cell line 5673 (grade II; [32]) by blocking its release and examining whether in vitro siRNA-mediated TGFBI suppression in these bladder cancer cells affects proliferation and migration.

## 2. Results

### 2.1. Antibody Array Analyses Identify TGFBI and PF4 as Biomarker Candidates in Urine

To identify candidate biomarker proteins for UC in urine, we performed filter-based hybridization assays with urine samples from hospital controls with pathologically confirmed urocystitis (*n* = 6) and low-grade de novo UC patients (*n* = 6; screening approach). Samples were carefully selected and matched for gender, smoking status and age, but differed in the absence/presence of UC. Evaluation revealed two promising biomarker candidates (TGFBI and PF4), and, compared to urological controls, approximately 12 and eight-fold higher levels of TGFBI and PF4 were found in the urine samples of UC patients.

### 2.2. Verification by ELISA Shows Better Performance of TGFBI Compared to PF4

**Figure 1:**
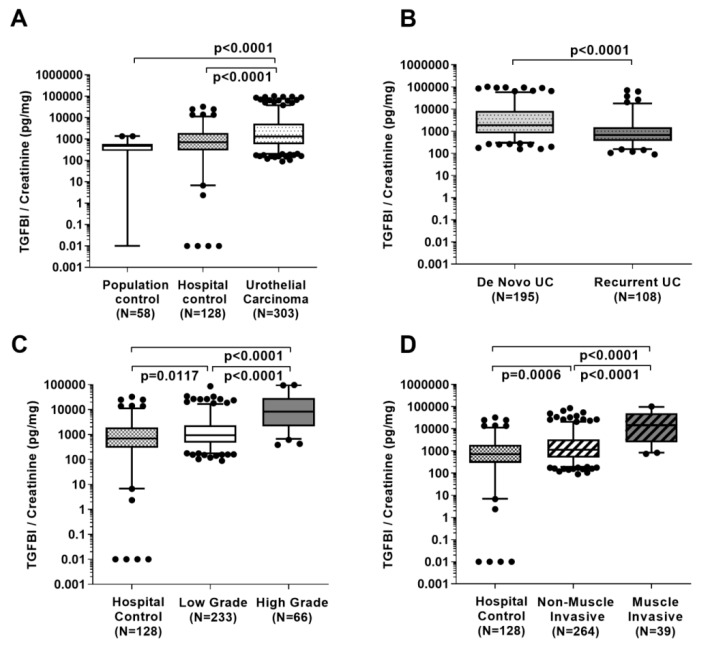
Creatinine-normalized TGFBI concentration in the urine of urothelial carcinoma (UC) patients, and hospital and population controls (**A**), in de novo UC patients and those with recurrent UC (**B**), in hospital controls compared to patients with low and high-grade UC (**C**) and compared to non-muscle invasive (≤pT1) and muscle invasive UC (>pT1) (**D**). Group differences were calculated by Wilcoxon rank-sum tests (non-parametric).

The verification of the antibody array results by using quantitative ELISA in a larger sample set, confirmed that median urinary TGFBI was higher in patients with UC in comparison to hospital and population controls (1321.0 versus 701.3 and 475.6 pg/mg creatinine, respectively; *p* < 0.0001; Figure 1A, Table 1). Within the group of de novo UC patients the median concentrations of TGFBI (1856.7 pg/mg) were higher than those with recurrent UC (658.2 pg/mg; *p* < 0.0001; Figure 1B; Table 1).

Median TGFBI concentrations were also elevated in low (943.1 pg/mg, *p* = 0.0117) and high-grade UC patients (8190.7 pg/mg creatinine, *p* < 0.0001; Figure 1C; Table 1) when compared to hospital controls, although are much more pronounced for high-grade UC. Furthermore, in muscle invasive UC patients median TGFBI levels were higher than in patients with non-muscle invasive UC and hospital controls (*p* < 0.0001; Figure 1D). However, TGFBI levels in recurrent UC did not differ significantly to those in hospital controls (*p* = 0.7465; Table 1).

**Figure 2:**
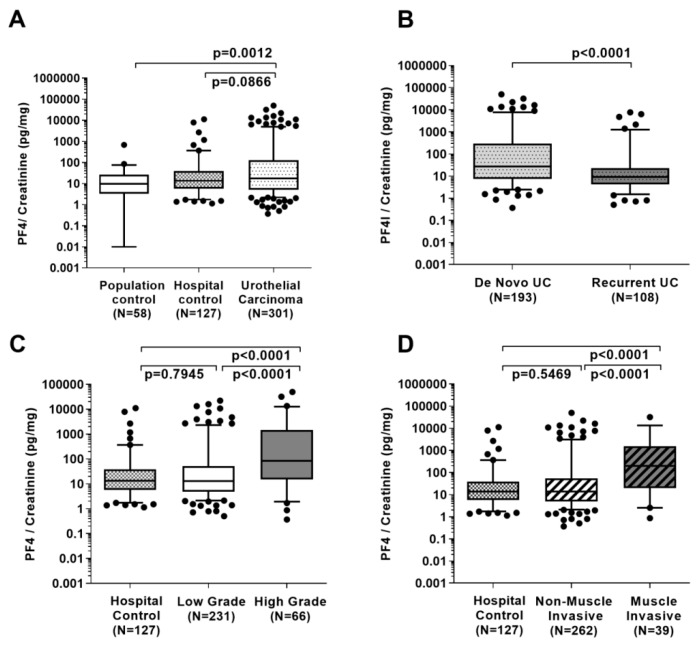
Creatinine-normalized PF4 concentration in urine of UC patients, and hospital and population controls (**A**), in de novo UC patients and those with UC history (**B**), in hospital controls compared to high and low-grade UC (**C**) and compared to non-muscle invasive (≤pT1) and muscle invasive UC patients (>pT1) (**D**). Group differences were calculated by Wilcoxon rank-sum tests (non-parametric).

Similar to TGFBI, median PF4 levels in patient urine, quantified by ELISA in the larger sample set, were higher in patients with UC (17.4 pg/mg creatinine) than in hospital controls (13.6 pg/mg) and population controls (median 9.6 pg/mg; Figure 2A; Table 1). However, the differences between UC patients and controls were far less pronounced compared to TGFBI and were only significant between patients with UC and population controls (*p* = 0.0012), whereas no difference could be observed between urothelial carcinoma patients and urologic controls (*p* = 0.0866). Median urinary PF4 levels were higher in patients with de novo UC than in those with recurrent UC (26.7 versus 9.2 pg/mg; *p* < 0.0001; Figure 2B; Table 1). PF4 levels were also larger in patients with high-grade UC (84.9 pg/mg) when compared to those with low-grade UC (12.8 pg/mg) and in patients with muscle invasive UC (201.9 pg/mg) when compared to those with non-muscle invasive UC (25.9 pg/mg) (Table 1; all *p* < 0.0001). No differences could be found for low-grade and non-muscle invasive types with hospital controls (*p* ≥ 0.5469; Figure 2C,D).

**Figure 3:**
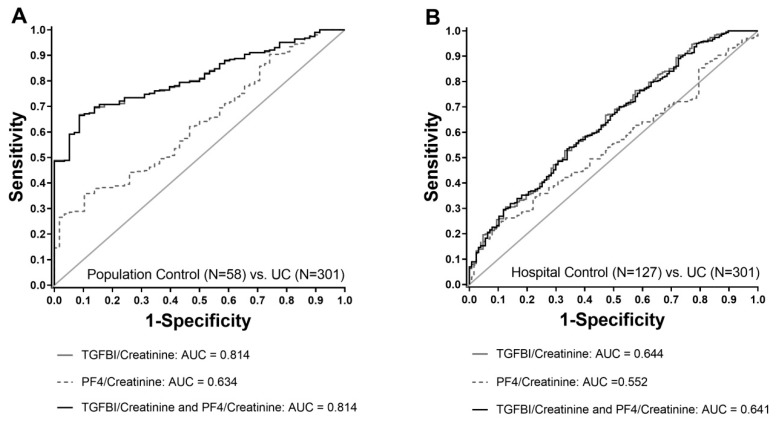
Receiving operator characteristic (ROC) curves of urinary TGFBI and PF4 alone and in combination. UC patients were compared with samples of the general population (**A**) and urologic controls from the hospital (**B**). For both curves, TGFBI alone and in combination with PF4, are nearly identical.

The improved performance of TGFBI compared to PF4 was confirmed by receiving operator characteristic (ROC) analyses. ROC analyses comparing UC patients and population controls revealed areas under the curves (AUCs) of 0.81 for TGFBI, whereas it was only 0.63 for PF4. For TGFBI, the sensitivity was 51.6% at a specificity of 95.0%. Combining TGFBI and PF4 did not improve ROC characteristics and the sensitivity and specificity remained completely unchanged (AUC 0.81, Figure 3A). Consequently, when comparing the population control versus UC patients, TGFBI displayed a sensitivity of 51.6% and a specificity of 95.0%, for both TGFBI alone and in combination with PF4. Similar results in terms of a better performance of TGFBI compared to PF4 were obtained when UC patients were compared to the hospital controls. The analysis revealed AUCs of 0.64 for TGFBI and 0.55 for PF4. The combination of TGFBI and PF4 did not further improve the results (AUC 0.64, Figure 3B). Based on these findings, we specifically focused on the evaluation of TGFBI as a biomarker candidate for UC.

### 2.3. TGFBI Predominantly Identifies Muscle Invasive and High-Grade UC

**Figure 4:**
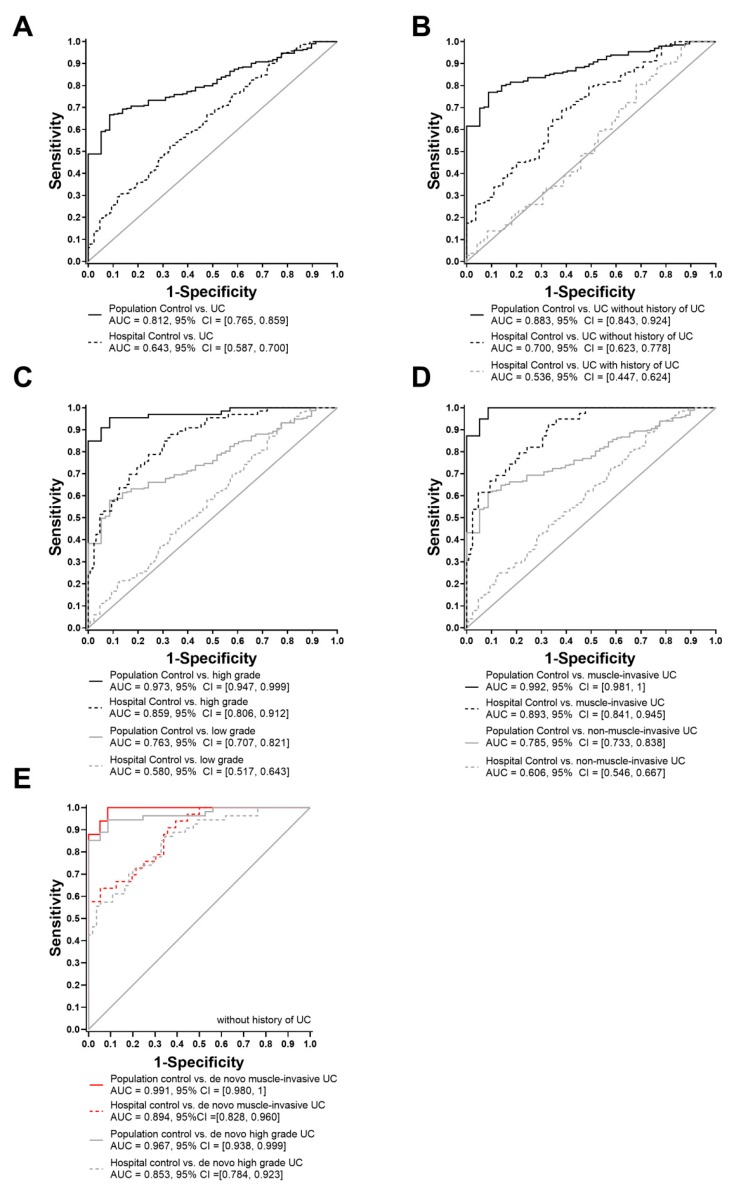
ROC curves of urinary TGFBI concentrations. The areas under the curves (AUCs) were determined by comparing different control groups to patients with (**A**) urothelial carcinoma, (**B**) de novo and recurrent UC, (**C**) low and high-grade UC, (**D**) non-muscle invasive UC (≤pT1) and muscle invasive (>pT1) UC (**E**) and de novo muscle invasive or high-grade UC.

Differences of TGFBI were always less pronounced between patients with UC and controls from the hospital compared to those between UC patients and population controls (Figure 4A). ROC analysis of patients with UC, distinguishing between de novo and recurrent UC, in comparison to both population and urological hospital controls, showed a higher sensitivity and specificity of TGFBI towards de novo UC patients (AUC 0.88 and AUC 0.70; Figure 4B). No differences, however, were observed between recurrent UC patients and hospital controls (AUC 0.54). Urinary TGFBI concentration was further evaluated for detecting high-grade or muscle invasive UC patients, because this is an essential biomarker context-of-use with regard to clinical decision making. Comparing grading and staging of the control groups versus UC patients resulted in a distinct higher specificity and sensitivity in high-grade (Figure 4C) and muscle invasive UC patients (Figure 4D). However, the overall best discrimination results with AUC levels of 0.86 and 0.89 were observed, again, in patients with de novo muscle invasive and high-grade UC, rather than in the respective patients with recurrent UC (Figure 4E).

### 2.4. Clinically Relevant Sample Characteristics Influence TGFBI Concentrations in Urine

The effects of potential modifying factors (e.g., age, gender, etc.) on TGFBI were tested by linear regression in the population controls, and within all study groups with multiple logistic regression analyses. No influence on urinary TGFBI could be found (Table 2). However, when we examined the influence of clinically relevant characteristics, such as stage and grade, on TGFBI levels we were able to confirm significantly higher TGFBI values in high-grade UC (exp(β) = 42.34, 95% CI 7.60–235.94) and in muscle invasive UC patients (exp(β) = 33.84, 95% CI 5.01–228.29) compared to the population controls (Table 2).

The multiple logistic regression analyses also confirmed that the marker showed increased performance in de novo UC compared to recurrent UC. In addition, the results revealed that, to a certain extent, the presence of leukocytes and erythrocytes alone or in combination affected the TGFBI concentration in urine (Supplementary Tables S1 and S2). This relationship was observed in all subgroups; however, when being positive for both leukocytes and erythrocytes, the median values of TGFBI in urine were also ten times higher in high-grade and/or muscle invasive UC patients (21,275 pg/mL) than in controls (2318 pg/mL; Supplementary Table S2)

### 2.5. TGFBI Is Required for Bladder Cancer Cell Proliferation

The elevated urinary TGFBI concentration in patients with high-grade UC suggests biological functions of this protein in bladder cancer. Therefore, we investigated the role of TGFBI on tumor cell proliferation by siRNA mediated gene silencing of TGFBI.

**Figure 5:**
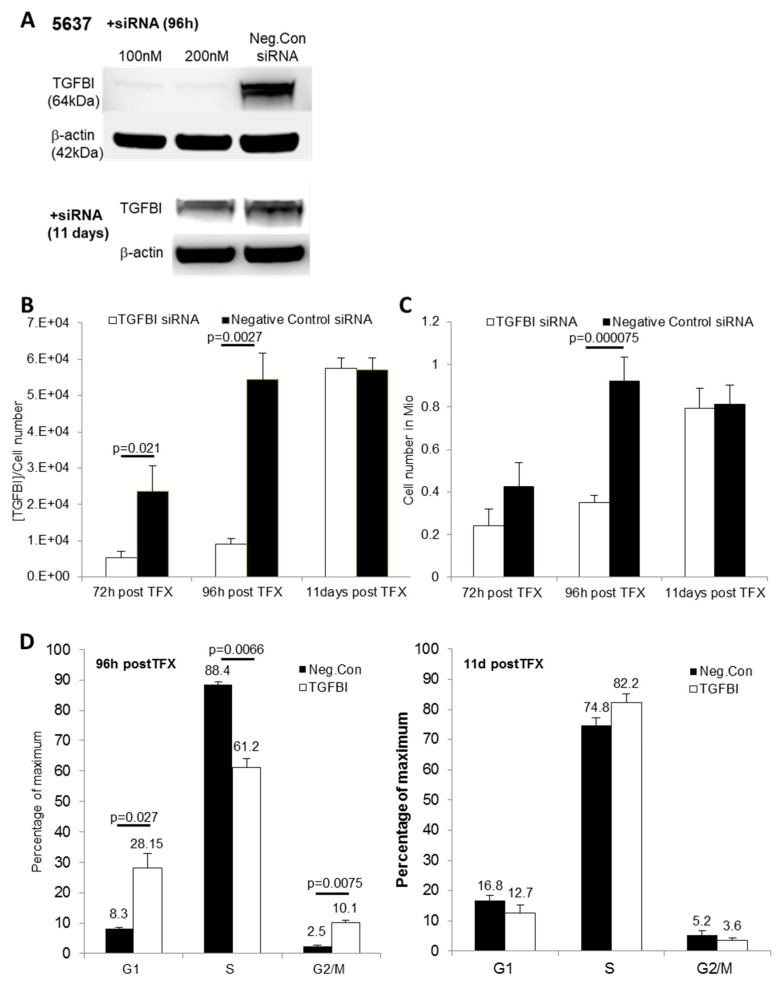
TGFBI siRNA transfected 5637 cells display a lower proliferation rate and an increased G1 cell cycle arrest. Altered protein expression in 5637 cells after successful knockdown with TGFBI siRNA compared to the non-targeting control 96 h and 11 days post-transfection (**A**), caused a significant reduction of TGFBI in the cell supernatant (**B**), which was accompanied by a decreased proliferation compared to the negative control, 72 h and 96 h post-transfection (**C**), and an elevated G1 cell cycle arrest (**D**). The percentage of cells in every cell cycle phase is displayed for each panel. Apoptotic cells were excluded. Error bars are displayed as +SD.

To verify that TGFBI was predominantly secreted by bladder cancer cells and not surrounding cells, we evaluated its secretion in immortalized normal cells of the bladder (UROtsa), and in addition, different human bladder cancer cell lines isolated from patients with muscle invasive bladder cancer (5637, J82). All cells secreted large amounts of TGFBI. Because 5637 cells proved to be most suitable for transient transfection compared to UROtsa and J82, we performed all experiments with this cell line. The 5637 cells were transiently transfected either by a negative control or TGFBI siRNA. Efficient TGFBI silencing was verified by western blot analysis (Figure 5A). Knockdown of TGFBI resulted in a significant decrease of TGFBI concentration in the cell supernatant in comparison to negative control (Figure 5B). The knockdown was accompanied by a drastic decline of cell growth after 96 h (4 days; *p* < 0.00007; Figure 5C). We also examined cell cycle progression of cells transfected with TGFBI-siRNA in comparison to negative control cells 4 and 11 days after transfection (Figure 5D). On day 4 (96 h) TGFBI-siRNA transfected cells significantly increased their G1 and G2/M phase populations by 19.9% (*p* = 0.0273) and 7.6% (*p* ≤ 0.0066) compared to negative control. Correspondingly, the S phase population decreased by 24% (*p* ≤ 0.0075). These significant effects on cell cycle were reversible after 11 days, when transient TGFBI expression returned to normal. Together, these results indicate that TGFBI-deficient 5637 bladder cancer cells have a disrupted cell cycle with, most likely, erroneous G1/S transitioning and S phase regulation. Thus, TGFBI secretion supports the growth of bladder cancer cells by increasing cell proliferation.

### 2.6. Elevated TGFBI Secretion in Bladder Cancer Cells Is Associated with Increased Cell Migration

**Figure 6:**
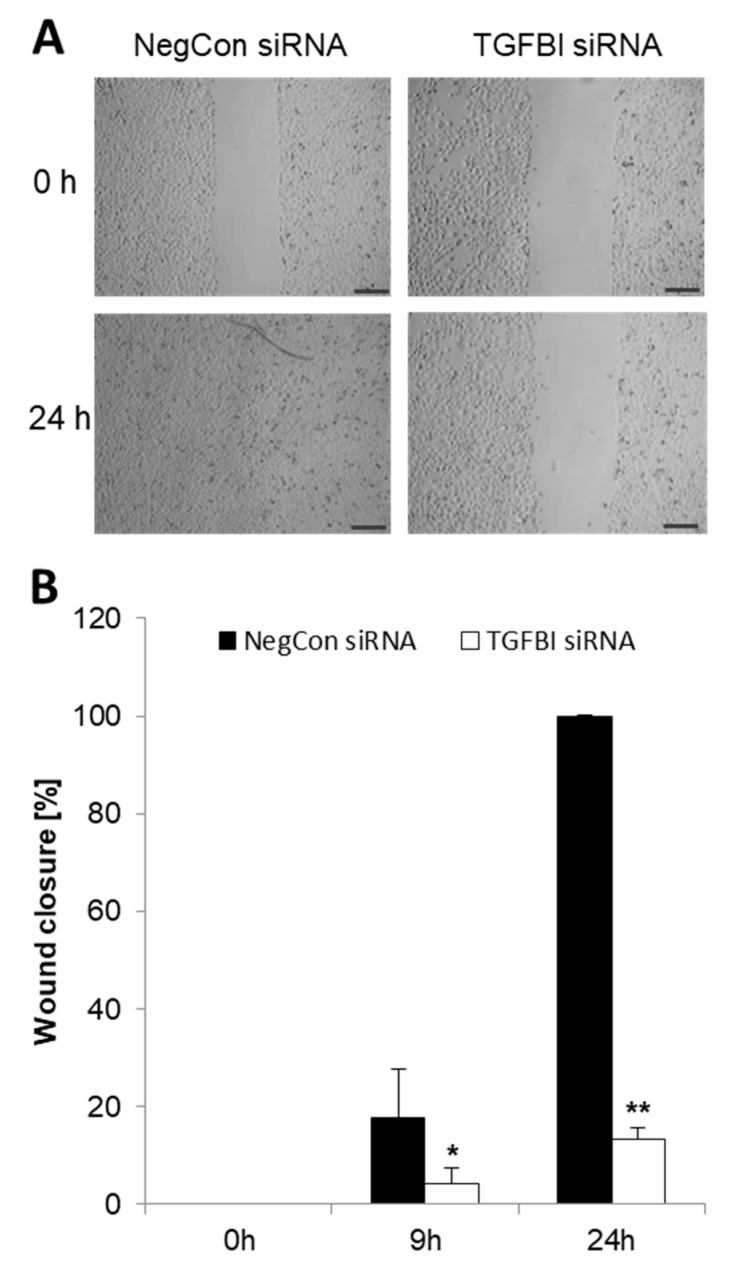
Wound closure assay 5637 cells. Representative images of 5637 scratch assays showing cells transfected with TGFBI siRNA and control siRNA, 0 h and 24 h after the wound was made (**A**). Accompanying quantification calculated as a percentage of wound closure in TGFBI siRNA-transfected cells compared to the negative control (**B**). Results are displayed as mean +SD (*n* = 3, * *p* < 0.026; ** *p* < 0.0009). Images were taken using a 4× objective; bar 200 µm.

To study a possible role of TGFBI in cell migration, 5637 cells were transfected with TGFBI-siRNA or negative control siRNA and then assessed by using a wound healing assay. Only 9 h after the wound was generated, negative control cells showed a significantly increased cell migration relative to TGFBI-deficient cells in terms of increased wound healing (Figure 6). After 24 h, we even observed an 87% delay of wound closure in 5637 cells transfected with TGFBI siRNA in comparison to control cells (*p* = 0.0009). This gap was still visible after 36 h. Hence, the expression and secretion of TGFBI by bladder cancer cells is critical for their migratory activity.

### 2.7. TGFBI Secretion Is Partially Induced via TGFβ Receptor 1 (TGFβR1)

**Figure 7:**
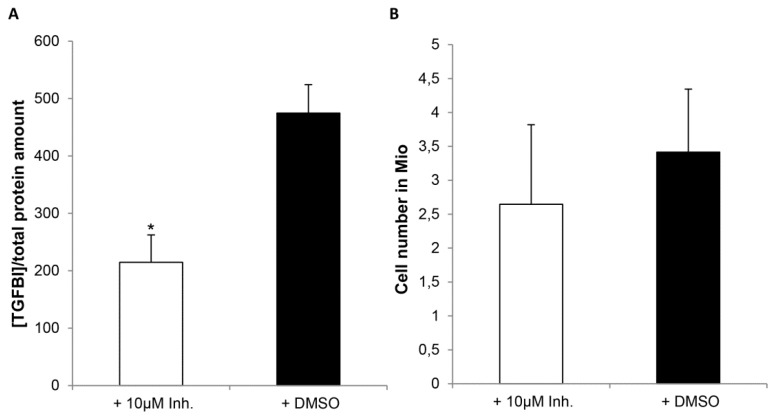
Inhibition of transforming growth factor-β (TGF-β) type I receptor by incubation of 5637 cells with 10 µM SB431542 for 48 h caused a significant decrease of TGFBI concentration in the cell supernatant ((**A**); * *p* ≤ 0.032), which was, albeit non-significant, accompanied by an inhibition of proliferation (**B**). Results are displayed as mean +SD (*n* = 3).

TGFBI was discovered in the lung adenocarcinoma cell line A549 as a cancer-associated gene induced by TGF-β1 [33]. To understand the signaling cascade leading to the expression and release of TGFBI in bladder cancer cells, we investigated the effects of the TGF-β-receptor type I inhibitor SB-431542 on 5637 cells. Incubation of the cells for 48 h with 10 µM inhibitor caused a significant decrease of TGFBI concentration in the cell supernatant (Figure 7A; *p* ≤ 0.032). This decline of TGFBI in the supernatant was accompanied by an inhibition of cell proliferation; however, the latter effect did not reach statistical significance (Figure 7B).

## 3. Discussion

In this study, we identified TGFBI and PF4 as protein biomarkers for identifying patients with high-grade UC and muscle invasive UC. Although PF4 at first, after screening with the protein array, seemed to represent a promising candidate, the subsequent confirmation by ELISA analyses and the evaluation of a larger sample set showed a much weaker performance compared to TGFBI. Because PF4 did not increase the performance of TGFBI in a combined model (PF4 plus TGFBI), we solely focused on the evaluation of TGFBI as a biomarker candidate for high-grade UC.

The observed preference of TGFBI in patients with de novo UC compared to controls was to be expected, due to the fact that de novo UCs are known to be larger in size compared to the corresponding recurrent UCs. Consequently, they secrete higher amounts of cancer-specific biomarkers. Similarly, the increased specificity and sensitivity of TGFBI could be observed in patients with muscle invasive UC and in those with high-grade UC (>pT1). These results were generated with a comparatively small number of tumor probes (*n* = 39 muscle invasive and *n* = 66 high-grade), so further validation is necessary. Moreover, de novo tumors showed a higher prevalence of high-grade UC (35%) than recurrent UC (12%). With the exception of a high rate of low-grade pT1 tumors, our collective is representative and in line of what is known from literature on the different rates of stages and grades of UC. The high rate of low-grade pT1 tumors most likely is due to a preselected patient population, a low risk collective where transurethral resection of the bladder (TURB) was the therapy of choice. An existing (focal) high-grade tumor within a low-grade tumor can, therefore, elude diagnosis, because TURB is inferior to cystectomy. The latter allows a complete histomorphological examination with exact localization of the tumor. However, missing cytomorphological atypia in pT1 tumors, thus classifying these tumors as low-grade, is also in line with their respectively (at the time of diagnosis) good prognosis [11]. Nevertheless, relapse and progression of pT1 tumors is high. Consequently, different staging and grading of the respective recurrent UCs, and thus overall worse prognoses, cannot be ruled out. Ultimately, to increase the statistical power regarding the value of TGFBI for UCs with poor outcomes, pooling studies and/or collectives is desirable. In order to assess the prognostic relevance of TGFBI, and this is generally important for biomarker studies, follow-up studies are necessary to assess the relationship between marker level and outcome (e.g., progression and disease-specific mortality).

The good performance of TGFBI for both high-grade and muscle invasive UCs specifically highlights its potential use as a biomarker for companion diagnostics. Patients with increased TGFBI values might benefit by an improved inspection of the bladder and the upper urinary tract, including photodynamic diagnostic and/or narrow band imaging, pyelography and even ureterorenoscopy. According to WHO guidelines 2004 [8], histological identification of a high-grade UC, necessitates a complete and precise pathological evaluation of all tissue samples to determine small areas of high-grade lesions inside defined samples.

Overall, a fast and simple ELISA readout of TGFBI in urine may sensitize both, the urologist and the pathologist to the potential presence of a high-grade UC. TGFBI companion diagnostics may be particularly valuable in such cases where histological sections cannot be completely evaluated (e.g., due to squeezing or cauterizing artefacts), or in terms of a verification analyses for a low-grade urothelial carcinoma. The latter is of particular importance, because the pathologist should not be allowed to miss a high-grade tumor.

The wide variation observed of TGFBI concentrations in the urine of both UC cases and controls, suggests that TGFBI cannot be used as a screening marker for UC diagnosis in the general population, independent of stage and grade. Even in some population controls, high TGFBI levels were observed. However, no detailed data on their current health status (e.g., other malignancies or inflammatory diseases) could be obtained. As with all newly identified biomarkers, TGFBI also needs verification in additional independent cohorts to cover a wide variety of patients with different diseases.

As mentioned, TGFBI identified high-grade and muscle invasive UC only. Moreover, multiple logistic regression analyses revealed that TGFBI levels were independent on gender, smoking status and age, but appeared to rise with the presence of leukocytes and erythrocytes alone and in combination (Supplementary Tables S1 and S2). This correlation between erythrocytes/leukocytes and the urinary TGFBI concentration has been observed in all subgroups (controls and UC patients). However, the median TGFBI level of all de novo high-grade and muscle invasive cases, which were positive for both erythrocytes and leukocytes, was ten times higher than in the corresponding (erythrocyte and leukocyte containing) controls, assuming that the high TGFBI concentration results from the specific tumor scenario. Nevertheless, the capability of reliably diagnosing high-grade UC in urine containing erythrocytes and leukocytes may need a more detailed investigation in future, e.g to establish separate cut-offs of TGFBI dependent on leukocyte and erythrocyte counts in urine. In addition, a more detailed understanding of the biological background leading to the increased TGFBI excretion in urine is necessary.

Although initial studies on biomarkers for the identification of UC were promising (including those which have been accepted by the FDA), so far no individual marker has been powerful enough to be implemented into clinical management. Instead, a compilation of several markers into diagnostic panels, separately addressing low-grade and high-grade disease or recurrence, appears the most promising way forward to improve risk stratification before transurethral resection of the bladder, and may specifically help to detect high-risk tumors. Especially in high-risk patients, extremely sensitive assays are required to not miss disease progression, tumor recurrence and persistence (e.g., pTis), as this disease can be fatal if detection fails. Overall, it might be worth to include TGFBI and our formerly identified CXCL16 in prospective studies as markers within a biomarker panel to detect high-grade and/or muscle invasive UC.

TGFBI is an extracellularly secreted matrix protein, proven to exist in normal and tumor cells. Secreted TGFBI interacts with other matrix proteins, such as collagen, fibronectin and laminin, thereby mediating proliferation, migration and cell adhesion; e.g., by interacting with integrins [26,27]. TGFBI has been shown to participate in differentiation, proliferation, tumor progression and metastasis [21,26,27,33]. In renal, pancreatic and colorectal cancers, TGFBI has been reported to act as a tumor promoter, and increased TGFBI expression has been observed [25,34,35]. Using a proteomics approach, TGFBI was demonstrated to be overexpressed in renal tumors with the worst prognosis and was significantly associated with oncological outcomes [36]. Shang et al. found that TGFBI effectively increases the adhesion, migration and invasion of A498 and ACHN renal cancer cell lines [37], further supporting its role in metastasis. The silencing of TGFBI in glioma and gastrointestinal cancer decreased local tumor growth and metastasis [38,39]. In line with these observations, we showed a tumor promoting function of TGFBI in bladder cancer in vitro, in terms of enhanced cell proliferation and migration. Our results are further supported by a study from Shang and colleagues, who demonstrated in RT112 and 253J bladder cancer cell lines [40], that siRNA-mediated low TGFBI expression significantly decreased proliferation, adhesion, migration and invasion. In contrast, overexpression of TGFBI in these bladder cancer cells significantly enhanced all those cellular functions. Although the molecular background responsible for the high expression of TGFBI in high-grade UC remains elusive, our in vitro results obtained in 5637 cells demonstrate that TGFBI clearly holds an independent and, most likely, unfavorable role in the progression of UC. Our results are corroborated by Zou and colleagues who, very recently, proved a significantly increased level of TGFBI mRNA and protein in the tissues of patients with muscle invasive bladder cancers and showed that a high TGFBI tissue expression was correlated with the histological grade and clinical stage [41].

In conclusion, we were able to identify TGFBI as an efficient biomarker candidate in the urine of patients with high-grade and/or muscle invasive UC. These patients may possibly need, due to less favorable prognosis, more aggressive therapeutic measures than patients with low-grade or non-muscle invasive UC. On the experimental level, we could demonstrate that TGFBI participates in the proliferation and migration of cancerous urothelial cells; therefore, specifically suggesting a critical role of this soluble protein in the tumorigenesis and progression of UCs with poor prognosis.

## 4. Materials and Methods

### 4.1. Subjects and Urine Collection

The spot urine samples of 431 patients who were suspected of having UC were collected at the Urologic Department of the Ruhr-University Bochum, Germany. Sampling was carried out before cystoscopy, and prior transurethral resection. Subsequently, two pathologists histopathologically examined all tissue samples and confirmed UC in 303 patients (52 women, 251 men) of whom 108 patients (35%) had recurrent UC and 195 patients (65%) had de novo UC, whereas 128 patients (36 women, 92 men) were cancer-free, but histology revealed an inflamed urothelium (urocystitis). This patient group with urocystitis was used as urologic hospital controls. The population control group was randomly selected from the residential registries with respect to the distribution of age, gender and catchment area in terms of distance to the hospital of the overall patient group (UC patients and hospital controls), and consisted of 58 healthy persons (without former UC, 11 women, 47 men) of the same catchment area (Table 1). Urine collection and processing was carried out with standard operating procedures (SOP). The study was ethically approved (number 3674-10; Ruhr University Bochum, approved on 9 March 2010 and all participants provided written informed consent.

### 4.2. Sample Preparation

Urine collection occurred in the morning. After centrifugation (10 min, 1700 g, 10 °C) urine samples were kept at –80 °C. The creatinine content was assessed according to Jaffé [42]. The measurement of leukocytes in urine (yes/no) and erythrocyte count (categorized into: negative, ~10, ~25–50 and ~150–250 per µL urine) were analyzed using Combur-Test^®^ sticks (Roche, Mannheim, Germany).

### 4.3. Antibody Arrays

An initial screening for biomarkers was performed using commercially available Proteome Profiler^TM^ antibody arrays (Human Soluble Receptor Array Kit, non-hematopoietic panel and Human Angiogenesis Array Kit; BioTechne, Wiesbaden, Germany). For analysis we used urine specimens from six patients with de novo UC and six hospital controls with histopathologically verified urocystitis. These specimens were carefully selected from the biobank at IPA after verification for sex, smoking status and age, thus only differing with respect to the absence/presence of UC. The antibody arrays were performed as described previously [19]. In short, membranes were blocked for 1 h at room temperature (RT), and then incubated with 500 µL untreated urine supernatant for 16 h at 6 °C. After washing (3×, 10 min, with washing buffer), membranes were left in the Detection Antibody Cocktail (2 h, RT). The washing step was repeated and then streptavidin-HRP (horseradish-peroxidase) was left on the membrane (30 min, RT). Captured proteins were visualized using chemiluminescence detection reagent (Pierce ECL, Thermo Fisher Scientific, Bonn, Germany). The signals produced are proportional to the amount of bound analyte. The image of the protein array was evaluated by LabImage 1D (Kapelan, Leipzig, Germany) and by quantifying the mean spot pixel densities from the array membrane. For semi-quantitative analysis of the changes in protein levels, the corresponding signals of every protein on the two different arrays (de novo tumor versus hospital control) were examined after background correction.

### 4.4. Cell Culture

The present study was performed on the human transitional bladder cancer cell line 5637 (European Collection of Animal Cell Cultures, Braunschweig, Germany). Cell identity was confirmed by STR analysis by comparing the profile to a reference STR profile stored at the Leibniz Institute DSMZ (German Collection of Microorganisms and Cell Cultures, Braunschweig, Germany). The 5637 cells were grown in RPMI medium (10% heat inactivated FCS and 1% penicillin-streptomycin). Cells were cultivated at 37 °C in a humidified 5% CO_2_ atmosphere. For inhibition experiments, we used transforming growth factor-β (TGF-β) Receptor Induced Kinase Inhibitor VI, (SB 431542; VWR, Darmstadt, Germany), said to suppress TGF-β-induced proliferation, migration and epithelial mesenchymal transition in several human cancer cell lines.

### 4.5. Enzyme-Linked Immunosorbent Assay

For TGFBI quantification in the supernatant of cells or urine specimens, we applied the human TGFBI DuoSet ELISA Kit, whereas for quantification of PF4 we employed the human PF4 DuoSet ELISA KIT (both BioTechne, Wiesbaden, Germany). Analyses were carried out according to the manufacturer’s protocol. Standardization by urinary creatinine concentration was obtained by dividing the TGFBI and PF4 concentration (pg/mL) of a particular urine sample by its corresponding creatinine level (mg/mL), such that normalized TGFBI and PF4 levels were reported in units of pg/mg creatinine.

### 4.6. siRNA Mediated Gene Silencing

The 5637 cells were transfected in 6-well plates (70% confluence) with SMARTpool siGENOME TGFBI and nonspecific control siRNA (Pool number 2) (GE Healthcare Dharmacon, Schwerte, Germany), and using Lipofectamine RNAi Max Transfection Reagent (Thermo Scientific, Dreieich, Germany). For transfection, lipofectamine reagent and respective siRNAs were separately diluted with FCS-free medium, incubated for 5 min at RT and then mixtures were combined. After 10 min at RT, siRNA was mixed with medium (without FCS) and pipetted into each well until a final siRNA concentration of 200 nM. After 4 h, 5637 cells were cultured in growth medium. After various time points, post-transfection supernatant was collected and kept frozen (–80 °C) until analyses. Additionally, cells were collected for protein isolation and BrdU assay performance. Successful transfection was controlled by immunoblotting against TGFBI in parallel to each experiment.

### 4.7. Protein Isolation and Immunoblotting

Cells, cultivated as a monolayer in a 6-well plate, were rinsed with ice-cold PBS and then scraped in 50 µL ice-cold RIPA-buffer, including a protease inhibitor (0.5%) (NuPage, all Sigma Munich, Germany). After freezing overnight, cells were disrupted using a motor-driven grinder (30–60 s), and then refrozen (–80 °C). The Pierce BCA Protein Assay Kit has been used for determining the protein concentration as described by the manufacturer (Thermo Fisher Scientific, Dreieich, Germany).

The quantitative expression of TGFBI protein was analyzed by immunoblotting. Therefore, 20 µg of every protein probe was heated in LDS sample buffer (10 min, 70 °C), and subsequently applied to gel-electrophoresis (NuPage, all Thermo Fisher Scientific, Dreieich, Germany). Protein transfer to a nitrocellulose membrane was carried out with an iBlot Gel transfer device, as described by the manufacturer. Antibody staining was performed using rabbit polyclonal anti-TGFBI (Proteintech, Manchester, UK) and a horseradish-peroxidase (HRP)-conjugated anti-rabbit antibody (Biocat, Heidelberg, Germany). For quantification of protein expression the applied amounts of protein were standardized by reprobing the membrane with a beta-actin mouse antibody and an appropriate secondary HRP-conjugated antibody (Sigma, Munich, Germany). The luminescence signal was induced with Pierce chemiluminescence blotting substrate (Thermo Fisher Scientific, Dreieich, Germany) and detected using a Hamamatsu C4742-98 system (Intas, Göttingen, Germany).

### 4.8. Wound Closure Assay

For wound closure/scratch assays, a culture insert (Ibidi GmbH, Planegg, Germany) was used. The 5637 cells, transfected with either TGFBI, or negative control siRNA, were added to the Ibidi chambers and left at 37 °C in 5% CO_2_. Seventy-two hours post-transfection, at time-point 0 h of the assay, the insert was gently removed, the cell patches were rinsed with 1x PBS and were then re-filled with growth medium. Cell migration was recorded by light microscopy (4× magnification). The experiments were performed in duplicates (*n* = 4). The scratch area was analyzed, employing CellSens software from Olympus (Hamburg, Germany).

### 4.9. BrdU Cell Cycle Assays

DNA synthesis was assessed by BrdU (5-bromo-2′-deoxyuridine) incorporation into siRNA-transfected 5637 cells. Cells were pulse-labeled with 10 μM of BrdU for 1 h at 37 °C, except to the non-pulsed control. Only actively proliferating cells incorporate BrdU into their DNA. After 1 h of pulse-labeling, the culture medium was exchanged by fresh medium. Eighteen hours after BrdU incubation, cells were resuspended in the BD Cytofix/Cytoperm Fixation/Permeabilization Kit (Becton Dickinson, Heidelberg, Germany). In short, cells were resuspended in Cytofix/Cytoperm solution, mixed vigorously, and then left in the dark (30 min, RT). After washing with BD Perm/Wash buffer, cells were transferred into medium containing 10% DMSO and 90% heat-inactivated FCS, and kept at –80 °C until usage. After washing (2× with BD Perm/Wash) cells were incubated for 1 h at 37 °C in PBS containing DNase. Subsequent to washing, cells were incubated with the anti-BrdU antibody (30 min, RT; Pharmingen, Germany). Afterwards, cells were washed again (BD Perm/Wash) and incubated in the dark with allophycocyanin (APC)-rat anti mouse antibody (30 min, RT). After an additional washing step, cells were incubated with heat-inactivated RNase (30 min, 37 °C). After addition of propidium iodide (1 mg/mL; Sigma, Darmstadt, Germany), samples were kept in the dark and directly analyzed on a FACS Canto flow cytometer (Becton Dickinson, Heidelberg, Germany). Each experiment was done in triplicate.

### 4.10. Statistics

Medians and the inter-quartile ranges (IQR) of the creatinine-corrected TGFBI and PF4 values are presented. The differences between groups were calculated with Wilcoxon rank-sum tests (non-parametric). ROC (receiver operating characteristic) curves for TGFBI/creatinine and PF4/creatinine were constructed and the areas under the curve (AUC, 95% confidence intervals) were determined. In order to analyze which modifying factors (patient group, age, gender, smoking status, urinary leukocytes, urinary erythrocytes and history of UC) were influencing the TGFBI level, we calculated a multiple linear model for the log-transformed marker with the potential confounders as risk factors in the group of the population controls. We also estimated the risk of having a high TGFBI/creatinine value of ≥1345.97 pg/mg creatinine (95th percentile (P95) in population controls) with multiple logistic regression analyses, using the aforementioned factors as independent variables, and the dichotomized TGFBI/creatinine value (cutoff 1345.97 pg/mg creatinine) as an outcome in the entire study population. SAS (version 9.4, SAS Institute, Cary, NC, USA) was used for statistical calculations, and *p*-values <0.05 were judged as statistically significant. Data were plotted by using GraphPad Prism software, version 5.0.4 (GraphPad Software, San Diego, CA, USA).

## Figures and Tables

**Table 1:** Distribution of creatinine-normalized TGFBI and PF4 concentrations (in pg/mg creatinine) corresponding to individual sample characteristics for TGFBI (*n* = 489) and PF4 (*n* = 486). IQR, interquartile range; *N*, sample number; ^$^ erythrocytes/µL urine; ^1^ two tumors represent nested variants with bland cytomorphology but potentially poor outcomes; ^2^ one tumor represented a nested variant; ^3^ all low-grade; ^4^ 71 out of 102 pT1 tumors (non-muscle invasive) were low-grade (69.6%); ^5^ 2 out of 39 muscle invasive tumors were low-grade (5.1%); ^6^ all high-grade, as per definition.

		TGFBI (pg/mg Creatinine)						PF4 (pg/mg Creatinine)					
		Population Controls (*N* = 58)		Urological Hospital Controls (*N* = 128)		Urothelial Carcinoma (*N* = 303)		Population Controls (*N* = 58)		Urological Hospital Controls (*N* = 127)		Urothelial Carcinoma (*N* = 301)	
		*n*	Median (IQR)	*n*	Median (IQR)	*n*	Median (IQR)	*n*	Median (IQR)	*n*	Median (IQR)	*n*	Median (IQR)
	Total	58	475.6 (282.6–600.0)	128	701.3 (292.3–1882.4)	303	1321.0 (565.2–5311.2)	58	9.6 (3.4–26.7)	127	13.6 (5.7–38.7)	301	17.4 (5.2–126.4)
**Age (years)**	<70	33	426.0 (287.7–595.6)	70	617.0 (240.5–1631.7)	118	977.2 (495.9–2479.5)	33	9.2 (3.4–20.6)	70	13.4 (5.7–27.4)	117	14.1 (4.8–58.0)
	≥70	25	498.2 (228.4–624.8)	58	919.3 (388.7–2496.3)	185	1608.4 (608.0–6411.4)	25	14.8 (3.9–30.4)	57	15.5 (5.8–72.4)	184	19.8 (5.4–195.4)
**Gender**	Men	47	426.0 (258.0–595.6)	92	762.5 (299.0–1816.5)	251	1293.1 (565.2–4575.9)	47	9.3 (3.3–21.4)	91	11.0 (5.4–29.7)	250	15.2 (5.0–83.6)
	Women	11	568.8 (335.2–660.8)	36	603.9 (178.3–2237.9)	52	1892.3 (548.8–6780.0)	11	22.9 (3.4–34.3)	36	18.9 (8.7–77.2)	51	44.7 (6.6–321.3)
**Smoking Status**	Non smoker	21	482.9 (283.1–660.8)	26	1189.1 (553.1–2639.8)	54	1530.6 (473.7–6244.2)	21	8.7 (3.3–30.8)	26	18.9 (6.4–42.7)	54	25.2 (5.6–131.2)
	Former smoker	29	426.0 (231.1–581.3)	59	540.4 (221.5–1516.5)	146	1385.7 (582.7–5321.9)	29	9.9 (4.5–22.0)	59	12.1 (3.8–44.2)	145	14.0 (4.8–86.1)
	Smoker	8	328.3 (236.3–571.0)	32	662.2 (437.8–2032.7)	80	1267.4 (642.2–3991.8)	8	11.7 (3.3–28.5)	31	11.0 (4.0–23.8)	79	17.8 (6.2–121.0)
	Missing	0		11	1131.7 (10.6–3190.7)	23	956.2 (480.1–6818.7)	0		11	20.6 (7.7–290.5)	23	16.5 (4.5–230.4)
**Leucocytes**	Negative	45	426.0 (258.0–595.6)	64	430.5 (184.2–775.9)	158	894.4 (463.0–2142.4)	45	9.3 (3.3–22.0)	64	13.4 (5.8–27.1)	156	11.8 (4.7–33.1)
	~25	5	600.0 (558.3–624.8)	27	954.4 (492.1–2137.9)	83	1369.1 (603.0–5621.6)	5	4.5 (0.0–16.9)	26	14.7 (5.3–25.3)	83	18.2 (4.9–86.1)
	~100	5	482.9 (289.0–614.3)	10	1545.5 (620.0–2639.8)	23	4413.7 (1608.4–11,104.3)	5	22.9 (21.4–27.8)	10	8.8 (3.4–42.7)	23	131.0 (8.8–1584.0)
	~500	1	581.3	23	2317.9 (819.6–6892.0)	33	26,403.6 (1667.0–62,186.3)	1	3.9	23	29.7 (6.1–126.2)	33	1568.2 (16.5–4228.9)
	Missing	2	324.8 (126.6–523.0)	4	837.7 (537.7–1068.2)	6	2291.7 (792.1–6741.3)	2	39.6 (4.7 - 74.5)	4	9.1 (6.4 - 68.8)	6	17.5 (5.0–76.0)
**Erythrocytes ^$^**	Negative-~10	52	482.6 (282.9–616.6)	78	456.1 (190.9–910.5)	122	612.2 (353.9–1136.3)	52	10.1 (3.9–27.0)	77	11.0 (5.5–22.9)	120	9.6 (4.2–20.9)
	~25–50	2	387.9 (335.2–440.7)	21	1159.1 (552.1–2794.7)	58	1176.0 (533.6–2703.4)	2	2.5 (1.6–3.4)	21	20.2 (5.7–36.1)	58	8.6 (4.2–25.9)
	~150–250	1	0.0	26	2549.8 (1516.5–6892.0)	118	5612.2 (1569.0–18,846.9)	1	3.3	26	59.3 (9.4–223.2)	118	207.1 (19.8–1584.0)
	Missing	3	523.0 (126.6–595.6)	3	670.6 (404.8–1004.7)	5	1931.9 (792.1–2651.4)	3	20.6 (4.7–74.5)	3	9.2 (3.8–128.3)	5	5.7 (5.0–76.0)
**UC History**	No	57	468.9 (282.6–600.0)	55	670.6 (435.0–2268.8)	195	1856.7 (789.3–8193.0)	57	9.3 (3.4–26.7)	54	13.7 (5.3–64.5)	193	26.7 (7.8–294.0)
	Yes	0		72	701.3 (267.8–1462.6)	108	658.2 (362.9–1508.7)	0		72	13.3 (5.8–27.4)	108	9.2 (4.3–23.0)
	Missing	1	482.9	1	13,648.1	0		1	22.9	1	321.0	0	
**Tumor Grading**	Low grade ^1^					233	943.1 (480.1–2244.0)					231	12.8 (4.8–52.4)
	High-grade					66	8190.7 (2142.4–26,933.5)					66	84.9 (15.0–1402.4)
	Missing					4	2429.2 (1331.2–4279.2)					4	18.1 (11–42.5)
**Tumor Staging**	pTa ^3^					160	680.0 (385.5–1446.5)					158	10.4 (4.3–33.0)
	pT1 ^2,4^					102	2387.7 (878.8–8193.0)					102	25.9 (6.6–292.4)
	pT2, pT2a, pT2b ^2,5^					39	14,411.7 (2479.5–50875.1)					39	201.9 (19.8–1572.5)
	pTis only ^6^					2	2249.3 (1503.6–2995.0)					2	12.3 (8.8–15.9)

**Table 2:** Influence of study group and group characteristics: odds ratios with 95% confidence intervals (CIs) for having a normalized TGFBI value ≥1345.97 pg/mg creatinine (P95 in population controls) determined by multiple logistic regression analyses (*N^+^*: number of participants with normalized TGFBI value ≥1345.97 pg/mg creatinine). Participants with missing data in one or more sample characteristics were rejected from analysis; odds ratios (Exp(β)) with the 95% confidence intervals (CI) were shown; *N^+^*: number of participants with normalized TGFBI value ≥1345.97 pg/mg creatinine). Model 1 and 2 differed with regard to their evaluated study groups.

Model 1					Model 2				
		*N* (*N^+^*)	Exp(β)	95% CI			*N* (*N^+^*)	Exp(β)	95% CI
**Study group**	Population control	54 (2)	1		**Study group**	Population control	54 (2)	1	
	Hospital control	113 (34)	7.43	(1.54–35.72)		Hospital control	113 (34)	7.83	(1.62–37.41)
	UC (low-grade)	213 (81)	8.22	(1.79–37.64)		Non-muscle invasive UC	243 (106)	10.20	(2.18–45.23)
	UC (high-grade)	58 (50)	42.34	(7.60–235.94)		Muscle invasive UC	32 (28)	33.84	(5.40–239.08)
**Gender**	Male	349 (132)	1		**Gender**	Male	353 (135)	1	
	Female	89 (35)	0.54	(0.25–1.19)		Female	89 (35)	0.57	(0.27–1.21)
**Age (years)**	<70	200 (60)	1		**Age (years)**	<70	201 (60)	1	
	≥70	238 (107)	1.37	(0.79–2.41)		≥70	241 (110)	1.58	(0.91–2.71)
**Leucocytes**	Negative	246 (61)	1		**Leucocytes**	Negative	248 (63)	1	
	~25	106 (47)	1.93	(1.01–3.67)		~25	107 (48)	1.82	(0.98–3.41)
	~100	35 (22)	3.29	(1.197–9.06)		~100	36 (22)	2.69	(1.01–7.14)
	~500	51 (37)	4.27	(1.68–10.90)		~500	51 (37)	3.91	(1.47–9.44)
**Erythrocytes**	Negative - ~10	231 (30)	1		**Erythrocytes**	Negative - ~10	234 (32)	1	
	~25–50	76 (30)	2.54	(1.30–4.95)		~25–50	77 (31)	2.56	(1.36–4.96)
	~150–250	131 (107)	12.40	(6.42–23.93)		~150–250	131 (107)	12.87	(6.79–24.49)
**Smoking**	Never	98 (39)	1		**Smoking**	Never	99 (40)	1	
	Former	225 (84)	0.77	(0.37–1.60)		Former	226 (85)	0.81	(0.40–1.66)
	Current	115 (44)	0.57	(0.25–1.28)		Current	117 (45)	0.62	(0.28–1.36)
**Former UC**	No	276 (123)	1		**Former UC**	No	280 (126)	1	
	Yes	162 (44)	0.40	(0.23–0.72)		Yes	162 (44)	0.38	(0.22–0.68)

## Supplementary Materials

Supplementary materials can be found at https://www.mdpi.com/1422-0067/20/18/4483/s1. Analysis of TGFBI–leukocytes and erythrocytes positive: Table S1. Numbers of de novo UC and controls with positive and/or negative leukocytes and erythrocytes. Table S2. TGFBI (median, IQR; pg/mg) in leukocyte and erythrocyte positive and/or negative de novo UC and controls.

## Abbreviations

AUC	area under curve
CI	confidence interval
FDA	Food and Drug Administration
H	Hour
HRP	horseradish-peroxidase
IQR	interquartile range
NMP22	nuclear matrix protein 22
PF4	platelet factor 4
PURE	Protein Research Unit Ruhr within Europe
RT	room temperature
ROC	receiver operating characteristic curves
SOP	standard operating procedure
TGFBI	transforming growth factor beta-induced
UC	urothelial carcinoma
