# Supplementary material for: TGFBI Protein Is Increased in the Urine of Patients with High-Grade Urothelial Carcinomas, and Promotes Cell Proliferation and Migration
Source: Int J Mol Sci. 2019 Sep 11;20(18):4483. doi: 10.3390/ijms20184483 (PMC6770034; doi:10.3390/ijms20184483)
Supplement: Supplementary file 1 [file ijms-20-04483-s001.pdf]

Supplementary Material for:

TGFBI Protein Is Increased in the Urine of Patients with High-Grade Urothelial Carcinomas, and Promotes Cell Proliferation and Migration

Kerstin Lang, Selcan Kahveci, Nadine Bonberg, Katharina Wichert, Thomas Behrens, Jan Hovanec, Florian Roghmann, Joachim Noldus, Yu Chun Tam, Andrea Tannapfel, Heiko U. Käfferlein, Thomas Brüning

- **Table S1.** Numbers of de novo UC and controls with positive and/or negative leukocytes and erythrocytes (p. 2)
- **Table S2.** TGFBI (median, IQR; pg/mg) in leukocyte and erythrocyte positive and/or negative de novo UC and controls (p. 2)

Table S1. Numbers of de novo UC and controls with positive and/or negative leukocytes and erythrocytes.

	Total	Leukocytes & erythrocytes unknown	Leukocytes & erythrocytes negative	Leukocytes positive, erythrocytes negative	Leukocytes negative, erythrocytes positive	Leukocytes & erythrocytes positive
De novo high grade*	54	3	4	0	15	32
De novo muscle-invasive*	32	1	2	0	8	21
Population controls	58	3	41	9	3	2
Hospital controls	56	1	14	7	13	21
All controls	114	4	55	16	16	23

*de novo high-grade and de novo muscle-invasive UC overlap in 31 cases.

Table S2. TGFBI (median, IQR; pg/mg) in leukocyte and erythrocyte positive and/or negative de novo UC and controls.

	Total	Leukocytes & erythrocytes negative	Leukocytes positive, erythrocytes negative	Leukocytes negative, erythrocytes positive	Leukocytes & erythrocytes positive
De novo high grade*	10,164 (1,995; 26,933)	1,669 (1,079; 2,502)	-	3,068 (1,371; 5,603)	21,275 (8,191; 59,638)
De novo muscle-invasive *	15,648 (2,384; 43,333)	1,669 (1341; 1,998)	1,543 (1,543; 1,543)	2,678 (1,102; 8,540)	26,523 (14,412; 67,905)
Population controls	476 (283; 600)	426 (258; 597)	558 (289; 614)	335 (0; 441)	630 (600; 661)
Hospital controls	736 (438; 2,293)	510 (178; 828)	492 (85; 801)	655 (435; 1,787)	2,324 (883; 6042)
All controls	560 (288; 916)	441 (231; 622)	523 (288; 620)	583 (242; 1,709)	2,318 (661; 6,042)

* de novo high-grade and de novo muscle-invasive UC overlap in 31 cases.
